# Sixty-one thousand recent planktonic foraminifera from the Atlantic Ocean

**DOI:** 10.1038/sdata.2018.109

**Published:** 2018-08-28

**Authors:** Leanne E. Elder, Allison Y. Hsiang, Kaylea Nelson, Luke C. Strotz, Sara S. Kahanamoku, Pincelli M. Hull

**Affiliations:** 1 Department of Geology and Geophysics, Yale University, Yale 06520, USA; 2 Department of Bioinformatics and Genetics, Swedish Museum of Natural History, Stockholm 8109, Sweden; 3 Biodiversity Institute and Department of Ecology and Evolutionary Biology, University of Kansas, Kansas 66045, USA; 4 Department of Integrative Biology and Museum of Paleontology, University of California, Berkeley, CA 94720, USA

**Keywords:** Palaeoecology, Biodiversity, Macroecology, Biogeography, Palaeontology

## Abstract

Marine microfossils record the environmental, ecological, and evolutionary dynamics of past oceans in temporally expanded sedimentary archives. Rapid imaging approaches provide a means of exploiting the primary advantage of this archive, the vast number of fossils, for evolution and ecology. Here we provide the first large scale image and 2D and 3D shape dataset of modern planktonic foraminifera, a major microfossil group, from 34 Atlantic Ocean sediment samples. Information on more than 124,000 objects is provided, including general object classification for 4/5ths of the dataset (~ 99,000 objects). Of the ~ 99,000 classifications provided, more than 61,000 are complete or damaged planktonic foraminifera. Objects also include benthic foraminifera, ostracods, pteropods, spicules, and planktonic foraminifera test fragments, among others. This dataset is the first major microfossil output of a new high-throughput imaging method (*AutoMorph*) developed to extract 2D and 3D data from photographic images of fossils. Our sample preparation and imaging techniques are described in detail. The data provided here comprises the most extensive publically available archive of planktonic foraminiferal morphology and morphological variation to date.

## Background & Summary

Paleontology and evolutionary biology are in the midst of a revolution driven by the proliferation of three-dimensional imaging technologies^1,2^. Nano- to micro-CT scanning and synchrotron-based tomography provide powerful tools for addressing questions of ontogeny, morphology, ecology, and phylogeny through submicron-scale volumetric resolution of fossils^3–5^. Population-level studies of 3D-morphological evolution have remained relatively rare, however, due to the time and data intensive nature of these approaches^2^. To address this gap, we have developed a high-throughput approach for extracting 2D and 3D shape information from photographic images called *AutoMorph*^6,7^ and have used this technique to generate extensive image and shape data for modern planktonic foraminifera.

Planktonic foraminifera are mixotrophic protists with calcium carbonate tests found primarily in the sunlit layers of the global ocean^8,9^. Due to their abundant fossil record and importance in paleoceanographic research, planktonic foraminifera and other microfossil groups (i.e., coccolithophores, radiolarians, and diatoms) have been the focus of many (semi-)automated approaches for extracting information on factors like size, 2D shape, calcite thickness, and species^10–13^. Despite this long history of extensive imaging, there are few shared datasets consisting of the primary data (i.e., original images and measurements) of the many of millions of microfossil measurements and images made to date (see http://data.nhm.ac.uk//dataset/henry-buckley-collection-of-planktonic-foraminifera), likely due to difficulty of sharing large files in the past^14^. Even the growing number data aggregators and archives like iDigBio, MorphBank, MorphoBank, and Figshare, have remits and/or storage limitations that preclude the storage of large datasets like the one we described here. This data sharing gap is important because it precludes the data being re-used for other purposes, including documenting the range of morphological variation within planktonic foraminiferal species.

Here we provide an extensive image library of modern planktonic foraminifera, with accompanying 2D and 3D coordinate data and morphometric measurements from Atlantic Ocean core top sediment samples. Images of 61,849 complete and damaged planktonic foraminifera are provided along with accompanying 2D and 3D morphometric data for nearly all objects (i.e., 57,304 of the complete and damage planktonic foraminifera provided were successfully extracted for 2D and 3D shape). Images and shape data for another ~37,000 classified objects is also provided in categories including planktonic foraminiferal fragments, pteropods, ostracods, etc. (see Methods for further details). We have withheld the object identities for 1/5th of the entire sample set (24,846 of the ~124,000 total objects) so that these images can be used as the test set for automatic image recognition algorithms (i.e., machine learning).

We primarily sampled morphological variation in the North Atlantic for practical and theoretical reasons. The vast majority of the roughly fifty morphological species of extant planktonic foraminifera are found in all ocean basins and hemispheres^15,16^, with morphological and genetic differentiation across environmental gradients^9,17^. Thus, while the dataset presented here primarily describes North Atlantic variation, it should be broadly representative of global variation in community morphology. From a practical perspective, we sampled in the Atlantic in order to obtain the best-preserved fossils. The Atlantic Ocean has far more well-preserved, carbonate-rich deposits, due to younger (i.e., less acidic) bottom waters and shallower average depths than the Pacific and Indian Oceans^18,19^. Preservation was important to ensure that we captured variation in morphology arising primarily from processes acting on living, rather than dead^20,21^, foraminifera.

Fossils were imaged and shapes extracted using automated slide scanning and a high-throughput image processing pipeline (*AutoMorph*), developed in-house to rapidly extract 2D and 3D shape information from light microscopic images^6,7^. Because the *AutoMorph* pipeline is relatively new, we describe in detail our sampling and imaging protocols for samples dominated by planktonic foraminifera. Relevant usage notes for this dataset are also provided. The *AutoMorph* software is available and frequently updated on GitHub (https://github.com/HullLab), and the images and shape data are available on Zenodo (http://doi.org/10.5281/zenodo.165514). The publically available dataset presented here provides the most extensive images, 2D and 3D shape documentation of the range of morphological variation observed in recent planktonic foraminifera to date, and provides a baseline for considering variation in morphology across both time and space.

## Methods

### Sample Selection and Preparation

**Figure 1:**
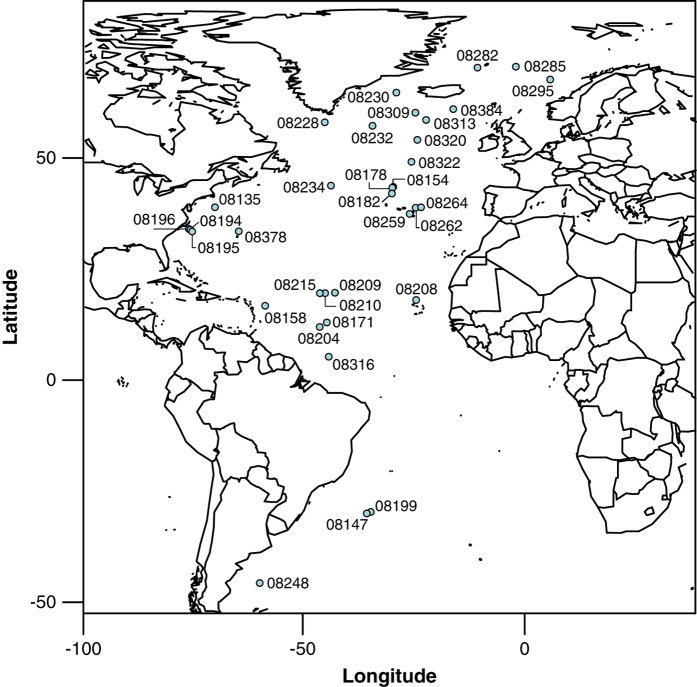
Map of sample locations. Sites investigated in this study, shown with Yale Peabody Museum (YPM) site numbers. Additional site information provided in Table 1.

Discerning the relative importance of environment, preservation, and biotic interactions on patterns observed in fossil assemblages often requires considering individual fossils in the context of their sedimentary environment and broader sample assemblage^22^. With this in mind, we imaged entire assemblages of fossils and sedimentary constituents from our 31 core top sediment sites from the North Atlantic and three core top sites from the South Atlantic (Fig. 1). Sites were chosen so as to span the five major planktonic foraminiferal faunal provinces identified by previous authors^15^, utilizing splits of core top fossil collections from B.H. Corliss (University of Rhode Island), R.D. Norris (University of California, San Diego), and M.J. Henehan (Yale University) (Table 1). Additional core top samples obtained from the Lamont-Doherty Core Repository for this study were dried, washed with deionized water over a 63μm sieve, and dried again at 50°C (see also Table 1). Sites and samples are from water depths above ~4000 m and had good to excellent preservation. To directly test for preservational effects, a few sites were selected along bathymetric depth transects (see Water Depth in Table 1). ‘Depth Interval' (depth below the sediment-water interface) varied from a minimum of 0-0.5 cm core depth to a maximum of 0-3 cm (Table 1), with broader depth ranges generally corresponding to a greater amount of geological time captured by the sample. Some of the core tops provided by B.H. Corliss had age estimates determined on the basis of benthic foraminifera oxygen isotopes^23^ (Table 1).

**Figure 2:**
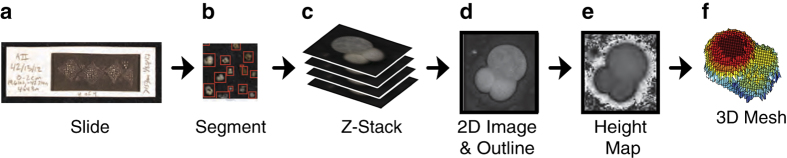
Abbreviated digitization workflow. The workflow includes slide preparation (**a**), imaging and object identification (**b**), isolation of object-specific depth slices (i.e., *z*-stack images) (**c**), and 2D (**d**) and 3D (**f**,**e**) shape extraction. Some images modified from ref. 7.

All core top samples were sieved to obtain the >150 μm fraction. The >150 μm fraction was then subsampled with a micropaleontological microsplitter down to ~5000 objects (primarily composed of planktonic foraminifera). Subsampled objects were arranged and lightly glued to plain black micropaleontological slides using a binocular stereo microscope (Fig. 2). Foraminifera were oriented with the umbilical side facing up, and fragments and ostracods were oriented with the concave side up. We aimed to mount ~1000 objects per slide, in order to prevent adjacent objects from touching, for a total of between 3–6 prepared slides for each subsample of ~5000 objects. In all, 155 slides were prepared from the 34 sites (Table 2 (available online only)).

### Imaging

Prepared slides were imaged using a 5-megapixel Leica DFC450 digital camera mounted on a Leica Microsystems DM6000M compound microscope with a drive focus and motorized *x-y* scanning stage. The microscope system is controlled by Surveyor Software (Version 7.0.1.0, Objective Imaging Ltd) run on a Dell computer (3 TB Solid-State Drive, 3.7 GHz processor) coupled to an OASIS-blue 3 Stage Controller (Objective Imaging Ltd) and a 5-megapixel Leica DFC450 digital camera. Three slides were prepped and scanned at a time using Surveyor’s multi-slide scanning mode (i.e., Navigator mode), which allows for multiple user defined scanning regions and variable background heights. Under our imaging pipeline, every slide scan generates a stack of raw slide images (called planes) at different *z*-axis heights. The number of planes per slide depends on the *z*-step size and the *z*-range (i.e., vertical extent of the volume imaged). All slides in this study were imaged with a *z*-range of 950 μm and a *z*-step size of 31.1 μm. Every slide region defined in Navigator was imaged and saved as a series of BigTIFFs: one BigTIFF for every z-plane through the slide and a single extended-depth-of-field (EDF) composite image. The BigTIFF image format is an extension of the more common Tiff file format, but is designed for large images (>4GB). In this study, all slides were imaged with a 5x objective and illuminated with dark field lighting.

### AutoMorph (automated morphometric post-processing)

Imaged slides were processed with the *AutoMorph* software package (http://github.com/HullLab), a bioinformatics pipeline designed to segment individual objects from light images and extract 2D and 3D shape information^6,7^. There are four major routines in *AutoMorph*: segment, focus, run2dmorph, and run3dmorph. The first two routines (segment and focus) identify all the unique objects in a raw image (i.e., a scanned slide), extract and label all the objects from the raw images, and save the individual *z*-slices in unique directories, generating a single best 2D extended depth of focus (EDF) image for each object. Two different programs can be used to generate the best 2D EDF: the commercially available *Zerene Stacker* (ver. 201404082055) and the open source *ImageJ*. We generated all 2D EDFs with *Zerene Stacker* because it consistently produced better EDF images. The second two routines (run2dmorph and run3dmorph) extract shape coordinates and basic measurements in 2D and 3D, respectively, along with images of the 2D and 3D shape extraction for quality control. This software package is freely available on GitHub (http://github.com/HullLab) and the methods are described in detail in two publications^6,7^. Because we developed *AutoMorph* to generate this data set, code updates were made over the course of the project. Code versions and processing dates are listed in Table 2 (available online only) to track these revisions. *AutoMorph* is adapted to run on local computers and clusters, and this dataset was generated using both.

**Figure 3:**
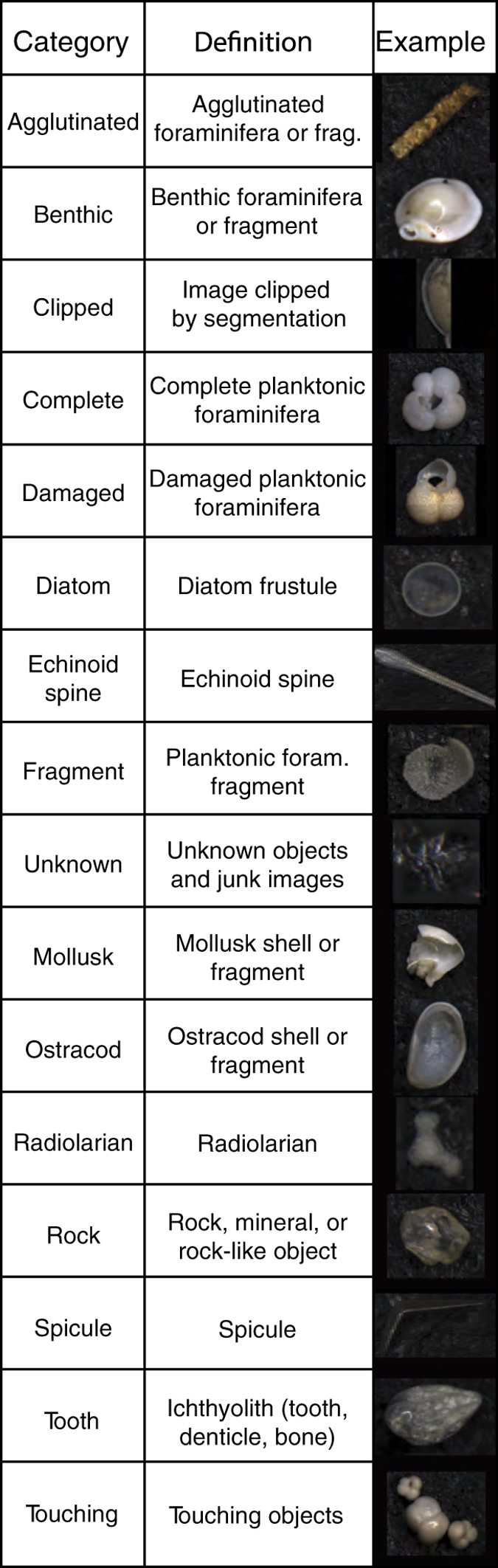
Illustrated classification categories with expanded definitions.

Once slide images were processed, all unique objects were identified by human observers (PMH and LEE) to one of 16 categories (Fig. 3) using classify (available at http://github.com/HullLab). classify is a custom-made application for viewing and assigning general object information to images (Supplementary Table 1). Supplementary Table 1 lists the classification (Object Type) of all objects (listed by Object ID) by slide (YPM Catalog Number), along with classification confidence (Certainty). In total, 124,230 unique objects from 155 slides were segmented and classified (Supplementary Table 1, and Table 3). In Supplementary Table 1, we provide information for 4/5 ths of the sample set (99,384 objects). The remaining 1/5 th of the sample set (i.e., 24,846 objects) is listed only as ‘withheld’ so that these objects can be used to test machine learning algorithms trained on the dataset provided here. Table 3 indicates the number of objects in each object category by sample site.

## Data Records

Metadata and images are provided for all 124,230 objects in the data set, with 2D and 3D shape information successfully extracted from 109,198 objects. Of the 61,849 complete and damaged planktonic foraminifera identified here with images and metadata, 57,304 also have accompanying 2D and 3D shape information and an additional 2,500 have 2D shape information only. The tables in this data report provide relevant metadata, summary statistics, and details on the technical validation of measurements. Sample identity, location, source, and handling information is provided in Table 1 and visualized in Fig. 1. Table 2 (available online only) provides relevant image processing information for the shape extraction pipeline in *AutoMorph*. The basic workflow is likewise shown in Fig. 2. The sixteen major categories used for classification are listed in Fig. 3 and the object classification results are provided in Supplementary Table 1 and summarized in Table 3. Because 2D and 3D shape and size information was extracted automatically, Table 4 provides technical validation for ten objects measured by stage micrometer, in *ImageJ*, and with all the various *AutoMorph* code versions used in this study. The data products of this research are all available on Zenodo (Data citation 1). The Zenodo data citation includes nine distinct data types uploaded as 13 distinct files and includes:

i) slide_images.tar.gz: 155 slide images with boxed objects identified by segment

ii) edf_images.tar.gz: 124,230 EDF images; i.e, one image for each object in the dataset

iii) obj_zstacks_part1.tar.gz and obj_zstacks_part2.tar.gz: combined, parts 1 & 2 contain 124,230 object specific subdirectories each with the original zstack images for the specific object

iv) 2d_outline_check.tar.gz: 113,847 EDF images of the objects successfully extracted for 2D outlines (included for quality control) and one text file (unextracted_objects_2D.txt) listing the 10,384 objects with failed extractions

v) 2d_coordinates.tar.gz: 155 CSV files containing all extracted outline coordinates for each of the 155 slides imaged, a text file of failed 2D extractions (unextracted_objects_2D.txt), and a summary CSV file including coordinates for all extracted objects (all_coordinates.csv)

vi) shape_measurements.csv: 2D and 3D measurements for all 124,230 objects

vii) metadata_tables.tar.gz: Tables 1, 2, 3, 4 and Supplementary Table 1 from this contribution, detailing the sample set metadata (i.e., site, sample, object information, and summary statistics about the dataset)

viii) 3d_pdfs_part1.tar.gz and 3d_pdfs_part2.tar.gz: combined, parts 1 & 2 contain 109,207 3D PDFs of objects successfully extracted for 3D shape (included for quality control) and one text file (unextracted_objects_3D.txt) listing the 15,023 objects with failed extractions

ix) 3d_obj_files_part1.tar.gz, 3d_obj_files_part2.tar.gz, and 3d_obj_files_part3.tar.gz: combined, parts 1,2, and 3 contain 109,207 3D shape coordinate files (.obj files) of objects successfully extracted for 3D shape and the text file of failed 3D extractions (unextracted_objects_3D.txt)

The first data product, the slide images of boxed objects, is also available in a low resolution version on the Yale Peabody Museum’s collection portal (http://collections.peabody.yale.edu/search/), under the division of Invertebrate Paleontology, by searching with the YPM collection number listed in Table 2 (available online only).

## Technical Validation

Technical validation occurred at a number of steps in the image processing pipeline, and included object selection, shape extraction, size measurements, and object classification.

### Object Selection

The *AutoMorph*
segment module saves a slide overview (a low resolution EDF) with each identified individual object boxed in red (Fig. 2; full sample set of boxed objects available in slide_images.tar.gz in data citation). To verify that all microfossils were identified and selected from each slide, we visually checked the boxed slide output. Image selection parameters in segment were adjusted as needed to optimize object selection. For a given set of image segmentation parameters, object selection is deterministic (i.e., the same objects are identified in the same order with every software run). The deterministic nature of the object selection software was verified by re-segmenting three slides twice and one slide three times and confirming the number and identity of objects. The number of objects outputted by segment were then cross checked with the number of objects outputted by all following modules (focus, run2dmorph, and run3dmorph) for each slide.

### Shape Extraction

2D EDF images of individual objects were generated by the focus module and this output was checked by eye for the first 100 objects in each slide to ensure proper image compositing (see edf_images.tar.gz in data citation). 2D and 3D shape extraction occurred along 2D outlines and 3D meshes of individual objects. The quality of 2D shape extraction was checked visually for the first 200 objects in each slide using outline-object overlays (see 2d_outline_check.tar.gz in data citation) and run2dmorph parameters were adjusted, when necessary, to optimize the efficacy of 2D outline extraction. Similarly, the quality and parameters of 3D shape extraction was checked visually using 3D PDFs of object meshes (see 3d_pdfs.tar.gz in data citation). Both run2dmorph and run3dmorph output lists of objects with failed outline (or mesh) extractions. These lists were examined for each slide to ensure that complete foraminifera were included and that specific species were not being disproportionately missed. When problematic (e.g., a large number of complete foraminifera failed to extract), the routines were re-run with different image extraction parameters to ensure the best possible 2D extraction. The same set of image extraction parameters yielded satisfactory results for 3D shape extractions of complete planktonic foraminifera from all samples.

### Size Measurements

**Figure 4:**
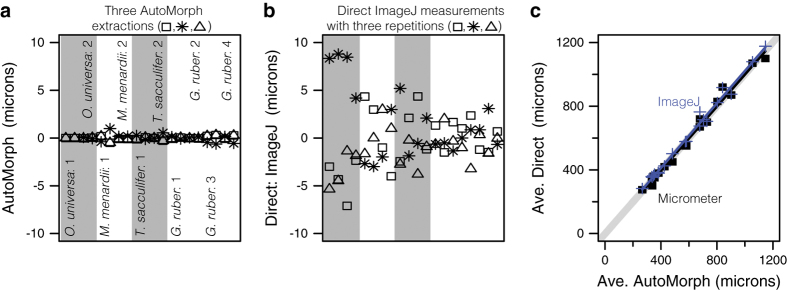
Technical validation of 2D size extraction. Repeated measurements of ten specimens (listed in Table 4) using three versions of *AutoMorph* (**a**) or three repeated hand measurements in ImageJ (**b**). Individual-specific data in (**a**) and (**b**) plotted as residuals of the mean individual-specific size measurement. Each individual (labelled in a) is represented by two columns of data (major axis and minor axis). Regression of micrometer measurements (black squares) and average *ImageJ* measurements (blue crosses) as a function of average *AutoMorph* size (**c**) did not significantly differ from the 1:1 line (light grey line).

The accuracy and reproducibility of 2D and 3D size extraction was confirmed with direct measurements. For run2dmorph, a calibration slide (IP.307866), containing ten complete planktonic foraminifera from four species, was used to check 2D size extraction (Table 4). This slide can be viewed in the YPM collections digital database (http://collections.peabody.yale.edu/search/). In total, ten complete planktonic foraminifera from four species were measured along their minor and major axes using a stage micrometer on a Leica S8APO microscope. The calibration slide was also segmented with each of the three code versions of the segment module of *AutoMorph*, and then processed through run2dmorph to obtain automated measurements of the major and minor axis for each individual foraminifer. The same individuals’ major and minor axis lengths were also measured in *ImageJ* using each of the three segment outputs. To do this, the ImageJ scale was set using the automatic scale bar added to the image label by segment, and the major and minor axes were drawn by hand. The three measurement types (run2dmorph, *ImageJ* and stage micrometer) were then compared (Table 4 and Fig. 4). Fig. 4a and b illustrate the relative reproducibility of the fully automated measurements (Fig. 4a: *AutoMorph*, three segment code versions) versus traditional *ImageJ* measurements (Fig. 4b: *ImageJ*). In both panels, object measurements are normalized to the mean measurement to highlight the variation between repeated measurements and the relative reproducibility of both approaches. *AutoMorph* (Fig. 4a) clearly outperforms hand measurements (Fig. 4b: *ImageJ*) in reproducibility, although both approaches have no significant difference between batches (*AutoMorph* one way ANOVA F(2,55)=0.0154, p=0.985; *ImageJ* one way ANOVA F(2,57)=0.00058; p=0.999). The small amount of variation that does exist between repeated *AutoMorph* measurements is due to a switch between a MATLAB code base (the original modules, segment versions 9-3-2014b and 10_27_2015) and a Python code base (segment version 6_17_2016). MATLAB code versions gave identical results, and all Python output was within 0.49 microns of the MATLAB output (Table 4). Repeated hand measurements in ImageJ had as much as a 16 micron difference between measurements. Importantly, all three approaches (*AutoMorph*, *ImageJ*, and measurement with a stage micrometer) provide the same average 2D measurements for foraminifera (Fig. 4c). Averaged *AutoMorph* output and stage micrometer measurements by specimen, as well as averaged *AutoMorph* output and *ImageJ* measurements by specimen were not significantly different (ANCOVA F(6,131)=0.036; p=1). Together, these tests indicate that *AutoMorph* provides accurate and reproducible 2D measurements of foraminifera. The accuracy and precision of 3D size extraction was previously assessed^7^ by comparing the height extraction with the length and width of spherical objects and by examining the effect of object orientation and imaging conditions on 3D mesh extraction and volume estimation (see ref. 7 for details). These tests indicated height extraction within 7.6% of the major and minor axis lengths for spherical objects.

### Object classification

Extensive spot checks of final EDF image classifications found object classification by human observers to be 99.95% accurate with different types of errors characterizing each classification category. The errors are described briefly here (category listed in quotes followed by a list of object-types included in error), with each classification category described in more detail in **Usage Notes**. Noted classification errors include ‘agglutinated’: clipped and unknown; ‘benthic’: clipped; ‘clipped’: mollusk and unknown; ‘complete’: damaged, clipped, and touching; ‘damaged’: complete; ‘echinoid spine’: unknown; ‘mollusk’: clipped and touching; ‘fragment’: unknown, mollusk, touching, and radiolarian; and ‘radiolarian’: clipped; ‘rock’: agglutinated. Chunks of consolidated sediment were generally poorly classified. The proper classification of a sediment chunk should be ‘rock’, a category which includes rock-like objects, but sediment chunks occurred in ‘agglutinated’, ‘touching’, ‘unknown’, and ‘rock’. Notably, as a category, ‘rock’ contained far more rock-like objects than actual lithic fragments. The occurrence of small foraminifera nested within complete, damaged and/or fragments of larger foraminiferal tests was similarly problematic. These combinations were assigned the classification of the larger object in cases where the small foraminifera were completely nested within the outline of the larger object. In cases where the small foraminifera obscured the outline of the larger object, the total image was classified as ‘touching’.

## Usage Notes

The splits of core top samples used in this study were, to our knowledge, unbiased by previous research efforts undertaken on the material, with exception to the benthic foraminifera. Many of the samples were picked for specific species of benthic foraminifera in the past, so all benthic foraminifera results should be considered as illustrative of some of the species present but not necessarily quantitative representations of their original abundance or full diversity in the samples. More generally, it is worth noting that most of the core top samples used here have a long collection history in other laboratories, so it is possible that selective sampling of some planktonic foraminifera or other species occurred in the past without our knowledge. Besides this effect, it is worth reiterating that the assemblage data provided here comes from death assemblages. In spite of visual evidence for good preservation in most of the core top samples included, selective dissolution of small-bodied and delicate species is known to begin even in the water column^20,21^, and the assemblages imaged are certainly time-averaged on the scale of hundreds to many thousands of years.

Objects that failed to properly extract for 2D and/or 3D shapes are listed in each of the appropriate data files (i.e., data citation files 2d_outline_check.tar.gz, 2d_coordinates.tar.gz, 3d_pdfs.tar.gz, and 3d_obj_files.tar.gz). Although we include all images extracted by segment in this dataset, do note that our initial sieve size was 150 microns. Although there are a number of objects smaller than 150 microns in this dataset, they are not representative of the abundance of this size category in the original sample. Rather, they are the rare objects that slipped through our size filter, and should be excluded for most applications. At least one ancient fossil appears in the core top data set. We have left this ancient fossil in as an indication of the level of cross-contamination in the lab (very low but potentially present). It is also possible that this stratigraphically out of place foraminifera was reworked in the sediments or introduced during handling in other labs. Regardless, users should remove this such outliers in species-specific applications.

Samples from YPM Sites IPE.08282, IPE.08285 and IPE.08295 were sized fractioned when received and different sized splits were taken from each size fraction. Here we described the post-processing that we carried out to insure that images from these samples accurately reflect species and size distributions at those sites. YPM Site IPE.08282 arrived in three sample jars containing, respectively, the 125-250 μm size fraction, the 250–315 μm size fraction, and the greater than 315 μm size fraction. The 125-250 μm size fraction was sieved over a 150 μm sieve and a 1/64th split was mounted on four slides (IP.308160, IP.308161, IP.308162, and IP.308163); a 1/32nd split of the 250-315 μm size fraction was mounted on two slides (IP.307847 and IP.307848); and a 1/32nd split of the greater than 315 μm size fraction was mounted on three slides (IP.307849, IP.307850 and IP.307851). This size-fractionated handling of these sites (Sites IPE.08282, IPE.08285 and IPE.08295) is problematic because it introduces a bias by over-representing certain size classes in the imaged object output. In the case of YPM Site IPE.08282 the largest two size fractions (the 250–315 μm and the greater than 315 μm size fractions) were over-represented by a 1/32nd split relative to the smallest size fraction (150–250 μm size fraction with a 1/64th split imaged). To correct for this bias, it was necessary to subsample the object output from these slides to properly represent the relative distributions of objects in the original sample.

More specifically, for IPE.08282 half the objects were randomly selected and discarded from the combined object list of IP.307847 and IP.307848 (the 250-315 μm size fraction) and from the combined object list of IP.307849, IP.307850 and IP.307851 (the greater than 315 μm size fraction), so that all size fractions contained a ~1/64th split of objects from the original site sample. For YPM Site IPE.08285, the largest two size fractions (the 250–315 μm and the greater than 315 μm size fractions) were over-represented by a 1/2nd split relative to the smallest size fraction (150–250 μm size fraction with a 1/16th split imaged). To obtain a 1/16th split across size fractions, one in every eight objects (12.5%) was randomly selected from the combined object lists of IP.307857 and IP.307858 (the greater than 315 μm size fraction) and from the combined object lists of IP.307859 and IP.307860 (the 250–315 μm size fraction). For YPM Site IP.08295, the largest size fraction (the greater than 250 μm size fraction) was over-represented by a 1/32nd split relative to the smallest size fraction (150–250 μm size fraction with a 1/256th split imaged). To obtain a 1/32nd split across size fractions, one in every eight objects (12.5%) was randomly selected from the combined objects in IP.307853 and IP.307854 (the greater than 250 μm size fraction). This data report includes objects *after* down-sampling and should be corrected for the bias introduced during slide preparation.

Each object was classified by a human observer according to one of sixteen categories (Supplementary Table 1), along with an indication of confidence in the classification: ‘very’, ‘somewhat’, and ‘not’. In a few classification categories, the confidence categories were used to indicate other attributes; these exceptions are explained below. Classification categories, listed in Fig. 3, were defined as follows. 'Agglutinated' indicates a complete agglutinated foraminiferal test, or some part thereof. Low confidence in this category (i.e., ‘agglutinated’, ‘not’) typically occurred when the agglutinated fragment was so small as to make it difficult to distinguish between an individual rock and individual foraminifera. ‘Benthic’ denotes any clearly identifiable piece of a benthic foraminifer (i.e., complete, damaged or fragment of a benthic foraminiferal test). Lower to low confidence (i.e., ‘somewhat’ or ‘not’) in the ‘benthic’ assignment arose when test fragments were too small to confidently assign or when individuals were too small or indeterminate to assign to either benthic and/or planktonic foraminifera categories. 'Clipped' indicates any image with at least one edge of the object clipped, with the exception of objects in the category ‘spicule’ as described below. ‘Complete’ indicates complete tests of planktonic foraminifera: a category that includes dirty tests (stained and/or visibly covered with some amount of sediment), but not tests that are broken or fragmented. The three confidence categories for ‘complete’ planktonic foraminifera were used in a non-standard way: i) ‘very’ indicates objects identified as complete planktonic foraminifera with high confidence; ii) ‘somewhat’ indicates all small bodied and juvenile individuals, where confident assignment to benthic or planktonic habitats was difficult; and iii) ‘not’ indicates planktonic species *Hirsutella scitula* and *Hirsutella theyeri* and similar looking benthic foraminifera. Damaged tests of planktonic foraminifera were classified as 'damaged' for all breaks, drill-holes, and damage assessed to affect less than around a third of the test. All cases of severe damage to planktonic foraminifera, including small planktonic foraminiferal fragments, were classified as ‘fragment’. The 'diatom' category contains diatom frustules, the 'echinoid spine' category contains echinoids spines, the ‘mollusk’ category contains mollusks, the ‘ostracod’ category contains ostracods, and the ‘radiolarian’ category contains radiolarians. In each of these (diatom, echinoid spine, mollusk, ostracod, and radiolarian), complete or large fragments of organisms were typically identified with greater confidence than small or out-of-focus pieces. Echinoid spines were confirmed as echinoid in nature by the match of the distinctive lattice structure in spine images with those of an immature echinoid in the YPM Invertebrate Zoology collection (YPM IZ.087653). The 'unknown' category contains non-target items, such as bits of background from the slide, fibres, and other unknown objects. Small pebbles, minerals, and other rock-like objects were categorized as 'rock'. Sponge spicules, categorized as ‘spicule’, were almost always clipped by the automated image segmenting routine. As a result, we included all clipped images of spicules in the category ‘spicule’ in spite of the incomplete nature of the image. All ichthyoliths (including fish teeth, shark dermal denticles, and other pieces of apatite) were categorized as ‘tooth’, with notably few actual teeth in this dataset. Small pieces of apatite and other ichthyoliths can be very difficult to identify, so many are likely categorized as ‘unknown’ or ‘rock’. Finally, when two or more objects touched, they were categorized as ‘touching’. Objects in direct or very near contact cannot be accurately extracted for 2D and 3D morphometrics.

## Additional information

**Publisher’s note**: Springer Nature remains neutral with regard to jurisdictional claims in published maps and institutional affiliations.

**How to cite this article**: Elder, L. E. *et al.* Sixty-one thousand recent planktonic foraminifera from the Atlantic Ocean. *Sci. Data* 5:180109 doi: 10.1038/sdata.2018.109 (2018).

## Supplementary Material



Supplementary Table 1

## Figures and Tables

**Table 1:** Metadata for collection and locality of sediment core samples used in this study. Sample Information.

YPM Site Number	IGSN Number	Research Vessal	Ship Abbreviation	Cruise ID	Leg	Station #	Core #	Coring Device	Depth Interval (cm)	Latitude	Longitude	Water Depth (m)	Source of Samples	Age (Sun et al. 2006)
IPE.08135	DSR00079D	Eastward	EA	EA04A-80			51	GC	0–1	38.9167	−69.7583	2993	Lamont	
IPE.08147	WHO0000C4	Chain	CH	CH-115	6	131	86	PG	0–2	−30.0017	−35.5617	2090	R.Norris-WHOI core top	
IPE.08154		Chain	CH	CH-82	8	49	19	PC	0–1	43.4883	−29.625	2630	R.Norris-WHOI core top	
IPE.08158	WHO0000A3	Chain	CH	CH-44	1	33	1	PC	0–3	16.7333	−58.45	4006	R.Norris-WHOI core top	
IPE.08171	WHO0001N9	Chain	CH	CH-75	2	29	19	PG	0–2	12.973	−44.568	3266	R.Norris-WHOI core top	
IPE.08178	WHO0002A9	Chain	CH	AII-82	8	51	21	PG	0–1	43.288	−29.83	2103	R.Norris-WHOI core top	
IPE.08182	WHO000206	Chain	CH	AII-82	6	28	8	PC	0–1	42	−29.9	2434	R.Norris-WHOI core top	
IPE.08194	WHO000664	Atlantis II	AII	AII-72	1	26	23	PG	0–2	33.825	−75.3	3204	R.Norris-WHOI core top	
IPE.08195	WHO000666	Atlantis II	AII	AII-72	1	27	24	PG	0–3	33.4483	−74.8917	3824	R.Norris-WHOI core top	
IPE.08196	WHO000662	Atlantis II	AII	AII-72	1	25	22	GC	0–1	34.0167	−75.6167	2942	R.Norris-WHOI core top	
IPE.08199		Atlantis II	AII	AII-60		10	10	PC	0–3	−29.66	−34.6667	1840	R.Norris-WHOI core top	
IPE.08204		Atlantis II	AII	AII-31	1	16	16	PG	0–3	11.9583	−46.1667	4217	R.Norris-WHOI core top	
IPE.08208	WHO0006T4	Atlantis II	AII	AII-42	1	2	2	PC	0–2	18.033	−24.45	3696	R.Norris-WHOI core top	
IPE.08209	WHO0006U3	Atlantis II	AII	AII-42	1	13	12	PC	0–2	19.667	−42.733	4043	R.Norris-WHOI core top	
IPE.08210	WHO0006U5	Atlantis II	AII	AII-42	1	15	14	PG	0–2	19.567	−44.95	3515	R.Norris-WHOI core top	
IPE.08215	WHO0006U7	Atlantis II	AII	AII-42	1	17	16	PC	0–2	19.5633	−46.13	2471	R.Norris-WHOI core top	
IPE.08228	DSR00078V	Maurice Ewing	EW	EW93-03	3		18	GC	0–0.5	58.02	−45.03	2358	Lamont	
IPE.08230	DSR00078R	Maurice Ewing	EW	EW93-03	3		4	GC	0–0.5	64.71	−28.91	1349	Lamont	
IPE.08232	DSR00078T	Maurice Ewing	EW	EW93-03	3		15	GC	0–0.5	57.24	−34.28	1923	Lamont	
IPE.08234	DSR00078P	Maurice Ewing	EW	EW93-03	3		34	GC	0–0.5	43.77	−43.64	2953	Lamont	
IPE.08248	DSR00079H	Vema	VM	VM-31	2	32	30	PC	0–1	−45.675	−59.678	1085	Lamont	
IPE.08259		Trident	TR	TR-121		37	37	PC	0–2	37.412	−25.902	2310	Bruce Corliss-URI	Holocene
IPE.08262		Trident	TR	TR-121		6	6	PC	top	38.793	−24.562	3770	Bruce Corliss-URI	Holocene
IPE.08264		Trident	TR	TR-121		7	7	PC	0–2	38.895	−23.338	3580	Bruce Corliss-URI	Holocene
IPE.08282		Meteor	M	M-10	3	625	MC218a	MC	0–0.5	70.33	−10.63	1710	Michael Henehan-Tuebingen	
IPE.08285		Meteor	M	M-10	3	637	MC218f	MC	0–0.5	70.54	−1.99	2795	Michael Henehan-Tuebingen	
IPE.08295		Meteor	M	M-21	5	317	MC323	MC	0–0.5	67.65	5.75	1411	Michael Henehan-Tuebingen	
IPE.08309		Vema	VM	VM-23		32	31	PC	0–1	60.183	−24.617	2178	Bruce Corliss-Lamont	Holocene
IPE.08313		Vema	VM	VM-27	6	117	108	PC	0–1	58.55	−22.2	2933	Bruce Corliss-Lamont	Holocene
IPE.08316		Knorr	KNR	KNR-142-2		A	78	KC	0–0.5	5.267	−44.133	3273	Bruce Corliss- WHOI	Holocene
IPE.08320	DSR000VL9	Vema	VM	VM-30	12	215	177	PC	0–1	54.067	−24.183	3433	Bruce Corliss-Lamont	Holocene
IPE.08322		Vema	VM	VM-29	9	188	182	PC	0–1	49.133	−25.5	3647	Bruce Corliss-Lamont	Holocene
IPE.08378	DSR00079L	Vema	VM	VM-20			248	PC	0–1	33.5	−64.4	1575	Lamont	
IPE.08384		Knorr	KNR	KNR-54	6	98	26	BC	top	60.995	−16.092	2435	Bruce Corliss- WHOI	Holocene
Metadata includes: Yale Peabody Museum (YPM) Site Number, International Geo Sample Number (IGSN; when available), and details of the research cruise and core recovery: Research Vessel, Ship Abbreviation, Cruise ID, Leg, Station Number, Core Number, Latitude, Longitude, and Water Depth at the sea floor. Coring Device indicates the approach used to obtain the samples and includes: gravity core (GC), piston core (PC), pilot gravity core (PG), Knight Core (KC), Box Core (BC), and multicore (MC). Although all samples were targeted as core top samples (i.e., from the surface of the sea floor), the precise depth in the core from which the sample came is indicated as the Depth Interval. Source is the researcher and/or repository sediment sample were provided from. Samples from B.H. Bruce Corliss had age data that were published in Sun *et al.* (2006) and are provided here.														

**Table 2:** Yale Peabody Museum sample information and digital processing history Sample digital processing history.

YPM Site Number	YPM Catalog Number	Segment Version	Processing Date: Segment	Run2dmorph Version	Processing Date: Run2dmorph	Processing Date:Run3dmorph
IPE.08178	IP.307625	2014-09-03a	15-04-2015	1.08	08-08-2016	8/23/2016-8/29/2016
IPE.08178	IP.307626	2014-09-03a	24-03-2015	1.08	08-08-2016	8/23/2016-8/29/2016
IPE.08178	IP.307627	2014-09-03a	16-04-2015	1.08	08-08-2016	8/23/2016-8/29/2016
IPE.08178	IP.307628	2014-09-03a	24-03-2015	1.08	08-08-2016	8/23/2016-8/29/2016
IPE.08316	IP.307630	2014-09-03a	21-09-2015	1.08	08-08-2016	8/23/2016-8/29/2016
IPE.08316	IP.307631	2014-09-03a	23-09-2015	1.08	08-08-2016	8/23/2016-8/29/2016
IPE.08316	IP.307632	2014-09-03a	12-10-2015	1.08	08-08-2016	8/23/2016-8/29/2016
IPE.08316	IP.307633	2014-09-03a	21-09-2015	1.08	08-08-2016	8/23/2016-8/29/2016
IPE.08316	IP.307634	2014-09-03a	21-09-2015	1.08	08-08-2016	8/23/2016-8/29/2016
IPE.08230	IP.307636	2014-09-03a	30-09-2015	1.08	08-08-2016	8/23/2016-8/29/2016
IPE.08230	IP.307637	2014-09-03a	30-07-2015	1.08	08-08-2016	8/23/2016-8/29/2016
IPE.08230	IP.307638	2014-09-03a	31-07-2015	1.08	08-08-2016	8/23/2016-8/29/2016
IPE.08230	IP.307639	2014-09-03a	02-10-2015	1.08	08-08-2016	8/23/2016-8/29/2016
IPE.08230	IP.307640	2014-09-03a	03-08-2015	1.08	08-08-2016	8/23/2016-8/29/2016
IPE.08204	IP.307642	12-02-2016	05-07-2016	1.08	08-08-2016	8/23/2016-8/29/2016
IPE.08204	IP.307643	2014-09-03a	16-06-2015	1.08	08-08-2016	8/23/2016-8/29/2016
IPE.08204	IP.307644	2014-09-03a	16-06-2015	1.08	08-08-2016	8/23/2016-8/29/2016
IPE.08204	IP.307645	2014-09-03a	16-06-2015	1.08	08-08-2016	8/23/2016-8/29/2016
IPE.08208	IP.307647	2014-09-03a	19-06-2015	1.08	08-08-2016	8/23/2016-8/29/2016
IPE.08208	IP.307648	2014-09-03a	26-03-2015	1.08	08-08-2016	8/23/2016-8/29/2016
IPE.08208	IP.307649	12-07-2016	01-08-2016	1.08	08-08-2016	8/23/2016-8/29/2016
IPE.08208	IP.307650	2014-09-03a	26-03-2015	1.08	08-08-2016	8/23/2016-8/29/2016
IPE.08208	IP.307651	2014-09-03a	26-03-2015	1.08	08-08-2016	8/23/2016-8/29/2016
IPE.08209	IP.307653	2014-09-03a	26-03-2015	1.08	08-08-2016	8/23/2016-8/29/2016
IPE.08209	IP.307654	2014-09-03a	26-03-2015	1.08	08-08-2016	8/23/2016-8/29/2016
IPE.08209	IP.307655	2014-09-03a	26-03-2015	1.08	08-08-2016	8/23/2016-8/29/2016
IPE.08209	IP.307656	2014-09-03a	26-03-2015	1.08	08-08-2016	8/23/2016-8/29/2016
IPE.08210	IP.307658	2014-09-03a	01-04-2015	1.08	08-08-2016	8/23/2016-8/29/2016
IPE.08210	IP.307659	2014-09-03a	01-04-2015	1.08	08-08-2016	8/23/2016-8/29/2016
IPE.08210	IP.307660	2014-09-03a	01-04-2015	1.08	08-08-2016	8/23/2016-8/29/2016
IPE.08210	IP.307661	2014-09-03a	07-04-2015	1.08	08-08-2016	8/23/2016-8/29/2016
IPE.08210	IP.307662	2014-09-03a	07-04-2015	1.08	08-08-2016	8/23/2016-8/29/2016
IPE.08215	IP.307664	2014-09-03a	09-04-2015	1.08	08-08-2016	8/23/2016-8/29/2016
IPE.08215	IP.307665	2014-09-03a	09-04-2015	1.08	08-08-2016	8/23/2016-8/29/2016
IPE.08215	IP.307666	2014-09-03a	10-04-2015	1.08	08-08-2016	8/23/2016-8/29/2016
IPE.08215	IP.307667	2014-09-03a	10-04-2015	1.08	08-08-2016	8/23/2016-8/29/2016
IPE.08215	IP.307668	2014-09-03a	17-09-2015	1.08	08-08-2016	8/23/2016-8/29/2016
IPE.08215	IP.307669	2014-09-03a	09-04-2015	1.08	08-08-2016	8/23/2016-8/29/2016
IPE.08215	IP.307670	2014-09-03a	09-04-2015	1.08	08-08-2016	8/23/2016-8/29/2016
IPE.08199	IP.307671	12-02-2016	05-07-2016	1.08	08-08-2016	8/23/2016-8/29/2016
IPE.08199	IP.307672	2014-09-03a	13-04-2015	1.08	08-08-2016	8/23/2016-8/29/2016
IPE.08199	IP.307673	2014-09-03a	13-04-2015	1.08	08-08-2016	8/23/2016-8/29/2016
IPE.08196	IP.307675	2014-09-03a	24-08-2015	1.08	08-08-2016	8/23/2016-8/29/2016
IPE.08196	IP.307676	2014-09-03a	24-08-2015	1.08	08-08-2016	8/23/2016-8/29/2016
IPE.08196	IP.307677	12-07-2016	07-07-2016	1.08	08-08-2016	8/23/2016-8/29/2016
IPE.08196	IP.307678	12-07-2016	03-08-2016	1.08	08-08-2016	8/23/2016-8/29/2016
IPE.08194	IP.307680	2014-09-03a	09-09-2015	1.08	08-08-2016	8/23/2016-8/29/2016
IPE.08194	IP.307681	2014-09-03a	09-09-2015	1.08	08-08-2016	8/23/2016-8/29/2016
IPE.08194	IP.307682	2014-09-03a	09-09-2015	1.08	08-08-2016	8/23/2016-8/29/2016
IPE.08194	IP.307683	2014-09-03a	01-10-2015	1.08	08-08-2016	8/23/2016-8/29/2016
IPE.08195	IP.307685	2014-09-03a	29-09-2015	1.08	08-08-2016	8/23/2016-8/29/2016
IPE.08195	IP.307686	2014-09-03a	29-09-2015	1.08	08-08-2016	8/23/2016-8/29/2016
IPE.08195	IP.307687	2014-09-03a	29-09-2015	1.08	10-02-2017	2/15/2017-2/16/2017
IPE.08195	IP.307688	2014-09-03a	02-10-2015	1.08	08-08-2016	8/23/2016-8/29/2016
IPE.08158	IP.307690	2014-09-03a	28-08-2015	1.08	08-08-2016	8/23/2016-8/29/2016
IPE.08158	IP.307691	2014-09-03a	01-09-2015	1.08	08-08-2016	8/23/2016-8/29/2016
IPE.08158	IP.307692	2014-09-03a	02-09-2015	1.08	08-08-2016	8/23/2016-8/29/2016
IPE.08158	IP.307693	2014-09-03a	02-09-2015	1.08	08-08-2016	8/23/2016-8/29/2016
IPE.08171	IP.307695	2014-09-03a	17-09-2015	1.08	08-08-2016	8/23/2016-8/29/2016
IPE.08171	IP.307696	2014-09-03a	17-09-2015	1.08	08-08-2016	8/23/2016-8/29/2016
IPE.08171	IP.307697	2014-09-03a	24-09-2015	1.08	08-08-2016	8/23/2016-8/29/2016
IPE.08182	IP.307698	2014-09-03a	02-09-2015	1.08	08-08-2016	8/23/2016-8/29/2016
IPE.08182	IP.307699	2014-09-03a	03-09-2015	1.08	08-08-2016	8/23/2016-8/29/2016
IPE.08182	IP.307700	2014-09-03a	04-09-2015	1.08	08-08-2016	8/23/2016-8/29/2016
IPE.08182	IP.307701	2014-09-03a	04-09-2015	1.08	08-08-2016	8/23/2016-8/29/2016
IPE.08154	IP.307703	2014-09-03a	03-09-2015	1.08	08-08-2016	8/23/2016-8/29/2016
IPE.08154	IP.307704	2014-09-03a	04-09-2015	1.08	08-08-2016	8/23/2016-8/29/2016
IPE.08154	IP.307705	2014-09-03a	04-09-2015	1.08	08-08-2016	8/23/2016-8/29/2016
IPE.08154	IP.307706	2014-09-03a	08-09-2015	1.08	08-08-2016	8/23/2016-8/29/2016
IPE.08154	IP.307707	2014-09-03a	08-09-2015	1.08	08-08-2016	8/23/2016-8/29/2016
IPE.08147	IP.307709	2014-09-03a	21-09-2015	1.08	08-08-2016	8/23/2016-8/29/2016
IPE.08147	IP.307710	2014-09-03a	18-09-2015	1.08	08-08-2016	8/23/2016-8/29/2016
IPE.08147	IP.307711	2014-09-03a	18-09-2015	1.08	08-08-2016	8/23/2016-8/29/2016
IPE.08147	IP.307712	2014-09-03a	18-09-2015	1.08	08-08-2016	8/23/2016-8/29/2016
IPE.08147	IP.307713	2014-09-03a	18-09-2015	1.08	08-08-2016	8/23/2016-8/29/2016
IPE.08378	IP.307715	2014-09-03a	11-08-2015	1.08	08-08-2016	8/23/2016-8/29/2016
IPE.08378	IP.307716	2014-09-03a	17-08-2015	1.08	08-08-2016	8/23/2016-8/29/2016
IPE.08378	IP.307717	2014-09-03a	11-08-2015	1.08	08-08-2016	8/23/2016-8/29/2016
IPE.08378	IP.307718	2014-09-03a	11-08-2015	1.08	08-08-2016	8/23/2016-8/29/2016
IPE.08135	IP.307775	2014-09-03a	14-09-2015	1.08	08-08-2016	8/23/2016-8/29/2016
IPE.08135	IP.307776	2014-09-03a	15-09-2015	1.08	08-08-2016	8/23/2016-8/29/2016
IPE.08135	IP.307777	2014-09-03a	15-09-2015	1.08	08-08-2016	8/23/2016-8/29/2016
IPE.08135	IP.307778	2014-09-03a	15-09-2015	1.08	08-08-2016	8/23/2016-8/29/2016
IPE.08135	IP.307779	2014-09-03a	15-09-2015	1.08	08-08-2016	8/23/2016-8/29/2016
IPE.08135	IP.307780	2014-09-03a	15-09-2015	1.08	08-08-2016	8/23/2016-8/29/2016
IPE.08248	IP.307781	2014-09-03a	16-09-2015	1.08	08-08-2016	8/23/2016-8/29/2016
IPE.08248	IP.307782	2014-09-03a	16-09-2015	1.08	08-08-2016	8/23/2016-8/29/2016
IPE.08248	IP.307783	2014-09-03a	16-09-2015	1.08	08-08-2016	8/23/2016-8/29/2016
IPE.08248	IP.307784	2014-09-03a	16-09-2015	1.08	08-08-2016	8/23/2016-8/29/2016
IPE.08248	IP.307785	2014-09-03a	17-09-2015	1.08	08-08-2016	8/23/2016-8/29/2016
IPE.08248	IP.307786	2014-09-03a	17-09-2015	1.08	08-08-2016	8/23/2016-8/29/2016
IPE.08232	IP.307789	2014-09-03a	11-09-2015	1.08	08-08-2016	8/23/2016-8/29/2016
IPE.08232	IP.307790	2014-09-03a	11-09-2015	1.08	08-08-2016	8/23/2016-8/29/2016
IPE.08232	IP.307791	2014-09-03a	11-09-2015	1.08	08-08-2016	8/23/2016-8/29/2016
IPE.08234	IP.307793	2014-09-03a	01-10-2015	1.08	08-08-2016	8/23/2016-8/29/2016
IPE.08234	IP.307794	2014-09-03a	02-10-2015	1.08	08-08-2016	8/23/2016-8/29/2016
IPE.08234	IP.307795	2014-09-03a	02-10-2015	1.08	08-08-2016	8/23/2016-8/29/2016
IPE.08234	IP.307796	2014-09-03a	02-10-2015	1.08	08-08-2016	8/23/2016-8/29/2016
IPE.08234	IP.307797	2014-09-03a	02-10-2015	1.08	08-08-2016	8/23/2016-8/29/2016
IPE.08228	IP.307799	2014-09-03a	14-09-2015	1.08	08-08-2016	8/23/2016-8/29/2016
IPE.08228	IP.307800	2014-09-03a	14-09-2015	1.08	08-08-2016	8/23/2016-8/29/2016
IPE.08228	IP.307801	2014-09-03a	14-09-2015	1.08	08-08-2016	8/23/2016-8/29/2016
IPE.08228	IP.307802	2014-09-03a	14-09-2015	1.08	08-08-2016	8/23/2016-8/29/2016
IPE.08228	IP.307803	2014-09-03a	14-09-2015	1.08	08-08-2016	8/23/2016-8/29/2016
IPE.08309	IP.307805	2014-09-03a	25-08-2015	1.08	08-08-2016	8/23/2016-8/29/2016
IPE.08309	IP.307806	2014-09-03a	25-08-2015	1.08	08-08-2016	8/23/2016-8/29/2016
IPE.08309	IP.307807	2014-09-03a	27-08-2015	1.08	08-08-2016	8/23/2016-8/29/2016
IPE.08309	IP.307808	2014-09-03a	25-08-2015	1.08	08-08-2016	8/23/2016-8/29/2016
IPE.08309	IP.307809	2014-09-03a	26-08-2015	1.08	08-08-2016	8/23/2016-8/29/2016
IPE.08262	IP.307811	2014-09-03a	09-09-2015	1.08	08-08-2016	8/23/2016-8/29/2016
IPE.08262	IP.307812	2014-09-03a	09-09-2015	1.08	08-08-2016	8/23/2016-8/29/2016
IPE.08262	IP.307813	2014-09-03a	10-09-2015	1.08	08-08-2016	8/23/2016-8/29/2016
IPE.08262	IP.307814	2014-09-03a	10-09-2015	1.08	08-08-2016	8/23/2016-8/29/2016
IPE.08262	IP.307815	2014-09-03a	10-09-2015	1.08	08-08-2016	8/23/2016-8/29/2016
IPE.08259	IP.307817	2014-09-03a	25-09-2015	1.08	08-08-2016	8/23/2016-8/29/2016
IPE.08259	IP.307818	2014-09-03a	25-09-2015	1.08	08-08-2016	8/23/2016-8/29/2016
IPE.08259	IP.307819	2014-09-03a	25-09-2015	1.08	08-08-2016	8/23/2016-8/29/2016
IPE.08259	IP.307820	2014-09-03a	29-09-2015	1.08	08-08-2016	8/23/2016-8/29/2016
IPE.08259	IP.307821	2014-09-03a	29-09-2015	1.08	08-08-2016	8/23/2016-8/29/2016
IPE.08313	IP.307823	2014-09-03a	10-09-2015	1.08	08-08-2016	8/23/2016-8/29/2016
IPE.08313	IP.307824	2014-09-03a	10-09-2015	1.08	08-08-2016	8/23/2016-8/29/2016
IPE.08313	IP.307825	2014-09-03a	10-09-2015	1.08	08-08-2016	8/23/2016-8/29/2016
IPE.08320	IP.307827	2014-09-03a	27-08-2015	1.08	08-08-2016	8/23/2016-8/29/2016
IPE.08320	IP.307828	2014-09-03a	27-08-2015	1.08	08-08-2016	8/23/2016-8/29/2016
IPE.08320	IP.307829	2014-09-03a	27-08-2015	1.08	08-08-2016	8/23/2016-8/29/2016
IPE.08320	IP.307830	2014-09-03a	27-08-2015	1.08	08-08-2016	8/23/2016-8/29/2016
IPE.08264	IP.307833	2014-09-03a	24-09-2015	1.08	08-08-2016	8/23/2016-8/29/2016
IPE.08264	IP.307834	2014-09-03a	24-09-2015	1.08	08-08-2016	8/23/2016-8/29/2016
IPE.08264	IP.307835	2014-09-03a	24-09-2015	1.08	08-08-2016	8/23/2016-8/29/2016
IPE.08322	IP.307837	2014-09-03a	10-09-2015	1.08	08-08-2016	8/23/2016-8/29/2016
IPE.08322	IP.307838	2014-09-03a	10-09-2015	1.08	08-08-2016	8/23/2016-8/29/2016
IPE.08322	IP.307839	2014-09-03a	10-09-2015	1.08	08-08-2016	8/23/2016-8/29/2016
IPE.08322	IP.307840	2014-09-03a	10-09-2015	1.08	08-08-2016	8/23/2016-8/29/2016
IPE.08322	IP.307841	2014-09-03a	10-09-2015	1.08	08-08-2016	8/23/2016-8/29/2016
IPE.08384	IP.307843	2014-09-03a	11-09-2015	1.08	08-08-2016	8/23/2016-8/29/2016
IPE.08384	IP.307844	2014-09-03a	11-09-2015	1.08	08-08-2016	8/23/2016-8/29/2016
IPE.08384	IP.307845	2014-09-03a	11-09-2015	1.08	08-08-2016	8/23/2016-8/29/2016
IPE.08282	IP.307847	12-02-2016	05-07-2016	1.08	08-08-2016	8/23/2016-8/29/2016
IPE.08282	IP.307848	2014-09-03a	12-10-2015	1.08	08-08-2016	8/23/2016-8/29/2016
IPE.08282	IP.307849	2014-09-03a	12-10-2015	1.08	08-08-2016	8/23/2016-8/29/2016
IPE.08282	IP.307850	2014-09-03a	12-10-2015	1.08	08-08-2016	8/23/2016-8/29/2016
IPE.08282	IP.307851	2014-09-03a	19-10-2015	1.08	08-08-2016	8/23/2016-8/29/2016
IPE.08295	IP.307853	2014-09-03a	27-08-2015	1.08	08-08-2016	8/23/2016-8/29/2016
IPE.08295	IP.307854	2014-09-03a	09-10-2015	1.08	08-08-2016	8/23/2016-8/29/2016
IPE.08295	IP.307855	2014-09-03a	28-08-2015	1.08	08-08-2016	8/23/2016-8/29/2016
IPE.08285	IP.307857	2014-09-03a	09-10-2015	1.08	08-08-2016	8/23/2016-8/29/2016
IPE.08285	IP.307858	2014-09-03a	12-10-2015	1.08	08-08-2016	8/23/2016-8/29/2016
IPE.08285	IP.307859	2014-09-03a	09-10-2015	1.08	08-08-2016	8/23/2016-8/29/2016
IPE.08285	IP.307860	2014-09-03a	09-10-2015	1.08	08-08-2016	8/23/2016-8/29/2016
IPE.08285	IP.308158	12-07-2016	27-12-2016	1.08	31-12-2016	1/4/2017-1/6/2017
IPE.08285	IP.308159	12-07-2016	27-12-2016	1.08	31-12-2016	1/4/2017-1/6/2017
IPE.08282	IP.308160	12-07-2016	27-12-2016	1.08	31-12-2016	1/4/2017-1/6/2017
IPE.08282	IP.308161	12-07-2016	27-12-2016	1.08	31-12-2016	1/4/2017-1/6/2017
IPE.08282	IP.308162	12-07-2016	27-12-2016	1.08	31-12-2016	1/4/2017-1/6/2017
IPE.08282	IP.308163	12-07-2016	27-12-2016	1.08	31-12-2016	1/4/2017-1/6/2017
Yale Peabody Museum (YPM) database records for each slide imaged include the YPM Site Number, and YPM Catalog Number. *AutoMorph* module code versions and dates processes are provided (i.e., segment Version, Processing Date: segment, run2dmorph Version, Processing Date: run2dmorph, Processing Date: run3dmorph) as *AutoMorph* modules were revised over the period of sample processing.						

**Table 3:** Summary statistics of object classifications summarized by YPM Site Number.

YPM Site Number	# of YPM Catalog Numbers per Site	# of Agglutinated per Site	# of Benthic per Site	# of Clipped per Site	# of Complete per Site	# of Damaged per Site	# of Diatom per Site	# of Echinoid Spine per Site	# of Fragment per Site	# of Unknown per Site	# of Mollusk per Site	# of Ostracod per Site	# of Radiolarian per Site	# of Rock per Site	# of Spicule per Site	# of Tooth per Site	# of Touching per Site	# of Withheld per Site	# Categorized Per Site (excluding Withheld)	# of Objects Per Site (Categorized + Withheld)
IPE.08135	6	483	34	41	1087	257		2	1967	544	35		6	87			24	1093	**4567**	**5660**
IPE.08147	5		12	8	2948	418			536	20	5	1	1	1			25	1042	**3975**	**5017**
IPE.08154	5	20	12	22	2142	145			207	116	12		4	75			12	769	**2767**	**3536**
IPE.08158	4	9	9	2	1679	219			455	81	3		1	3			30	623	**2491**	**3114**
IPE.08171	3		5	5	614	59			50	40			1	2			9	202	**785**	**987**
IPE.08178	4	11	15	58	2338	175			332	359	45	4	1	172			36	859	**3546**	**4405**
IPE.08182	4	18	25	16	1634	196		1	403	175	148	4	16	2			203	737	**2841**	**3578**
IPE.08194	4	84	9	66	1076	184	2	10	277	284	36	4	52	18	5	2	47	561	**2156**	**2717**
IPE.08195	4	43	13	32	1560	153		4	384	258	6	4	28	2		1	42	580	**2530**	**3110**
IPE.08196	4	241	37	106	949	223		7	399	1144	56	17	37	56	4		114	883	**3390**	**4273**
IPE.08199	3		1	29	931	94			106	80	359	1	6	2			23	404	**1632**	**2036**
IPE.08204	4	2	14	40	1365	263			391	61	2		1	2			32	491	**2173**	**2664**
IPE.08208	5	2	9	136	1622	122			149	56	7	2	3	1			16	556	**2125**	**2681**
IPE.08209	4		27	28	1731	188			426	52				1			18	596	**2471**	**3067**
IPE.08210	5		6	84	2152	241			470	85	8	2	1				20	719	**3069**	**3788**
IPE.08215	7		12	324	1516	101			169	298	2102		1	3			72	1141	**4598**	**5739**
IPE.08228	5	56	20	124	2169	59			92	341	10	1	1	906	4		13	952	**3796**	**4748**
IPE.08230	5	1	28	94	2405	437		1	589	399	21	6	1	57	3	25	36	1064	**4103**	**5167**
IPE.08232	3	4	19	14	1920	128	20	3	168	82	4	2	21	47	2		35	601	**2469**	**3070**
IPE.08234	5	7	19	44	2242	125			332	200	11		3	1080			52	1082	**4115**	**5197**
IPE.08248	6	3	59	75	1641	173		1	1350	296	29	1	98	189	6	3	39	994	**3963**	**4957**
IPE.08259	5	29	12	61	1285	84			229	507	188	3	13	300	2	2	144	730	**2859**	**3589**
IPE.08262	5	17	18	4	1884	189			630	232	8	1	14	84			214	811	**3295**	**4106**
IPE.08264	3	2	3	5	966	74			253	116			1	12		1	14	391	**1447**	**1838**
IPE.08282	9	176	77	520	1869	223	1		297	366	4	1	2	67		1	119	969	**3723**	**4692**
IPE.08285	6	42	80	66	1718	140	6	1	180	93	18		7	13	12		117	613	**2493**	**3106**
IPE.08295	3	169	51	54	408	34	1	3	234	313	37	1	4	155		1	28	365	**1493**	**1858**
IPE.08309	5	384	4	36	2625	278	1		608	683	7	3	22	52	1		126	1173	**4830**	**6003**
IPE.08313	3	6		3	1506	72			119	46			4	2			17	424	**1775**	**2199**
IPE.08316	5	6	4	52	1506	138		1	192	47	110		6	2			20	488	**2084**	**2572**
IPE.08320	4	288		5	1316	96			392	217	5	1	28	15			56	551	**2419**	**2970**
IPE.08322	5	534	1	6	1473	114			278	72	2	1	27	61	1		94	638	**2664**	**3302**
IPE.08378	4	4	28	63	2372	287			214	1536	320		5	6		1	31	1282	**4867**	**6149**
IPE.08384	3	48	4	2	1426	85	1	2	174	59	9	1	14	14		1	33	462	**1873**	**2335**
**Column Totals**	**155**	**2689**	**667**	**2225**	**56075**	**5774**	**32**	**36**	**13052**	**9258**	**3607**	**61**	**430**	**3489**	**40**	**38**	**1911**	**24846**	**99384**	**124230**
Number of slides per site (# of YPM Catalog Numbers per Site), withheld object IDs per site (# Withheld per Site), categorized objects per site (# Categorized per Site (excluding Withheld)), and objects per site (# of Objects per Site (Categorized+Withheld)) listed in bold. Object classification counts summarized by objected type at the bottom as ‘Column Totals’.																				

**Table 4:** Technical validation of automated 2D measurements.

		Stage micrometer (μm)		segment ver: 9-3-2014b		segment ver: 10_27_2015		segment ver: 6_17_2016							
ImageJ (μm)		AutoMorph (μm)		ImageJ (μm)		AutoMorph (μm)		ImageJ (μm)		AutoMorph (μm)		ImageJ (μm)		AutoMorph (μm)	
Species	Specimen	Major Axis	Minor Axis	Major axis	Minor axis	Major axis	Minor axis	Major axis	Minor axis	Major axis	Minor axis	Major axis	Minor axis	Major axis	Minor axis
*Orbulina universa*	1	700	700	709.91	719.80	728.22	704.35	707.55	719.65	728.22	704.35	696.23	706.53	n/a	n/a
*Orbulina universa*	2	550	550	580.76	579.43	581.36	574.85	586.49	579.95	581.36	574.85	570.91	573.40	581.27	574.96
*Menardella menardii*	1	1070	830	1081.00	823.02	1054.04	803.73	1075.00	820.04	1054.04	803.73	1082.02	826.01	1054.62	802.24
*Menardella menardii*	2	1100	920	1174.05	916.00	1146.31	840.28	1178.03	921.00	1146.31	840.28	1179.01	914.01	1146.04	839.97
*Globigerinoides sacculifer*	1	870	720	878.01	764.51	902.28	678.28	877.70	762.17	902.28	678.28	870.06	766.15	901.88	678.51
*Globigerinoides sacculifer*	2	670	450	679.41	502.57	677.26	482.12	671.26	502.84	677.26	482.12	676.18	499.57	677.29	481.29
*Globigerinoides ruber*	1	360	275	364.62	281.02	347.14	266.60	362.64	284.51	347.14	266.60	364.59	283.51	346.92	266.76
*Globigerinoides ruber*	2	380	300	379.01	354.53	382.37	337.55	377.01	352.50	382.37	337.55	380.03	353.51	382.33	337.41
*Globigerinoides ruber*	3	380	340	387.59	358.13	379.73	334.67	382.00	359.67	379.73	334.67	383.50	357.59	380.41	335.62
*Globigerinoides ruber*	4	420	350	426.69	373.39	425.43	353.37	426.57	372.67	425.43	353.37	422.00	374.02	425.18	354.20
This table contains measurements of individual foraminifera from YPM Catalog Number IP.307866. Each individual foraminifer (identified by Species and Specimen) was measured along its minor and major axes with a stage micrometer on a Leica S8APO microscope, and in *ImageJ* using the scale bar added by the segment module of *AutoMorph*. Automated size measurements from run2dmorph are also provided as ‘AutoMorph μm’ for each foraminifer. In one instance, run2dmorph failed to extract the object outline as indicated by the n/a.															
